# Influence of *Clitoria ternatea* Flower Extract on the In Vitro Enzymatic Digestibility of Starch and Its Application in Bread

**DOI:** 10.3390/foods7070102

**Published:** 2018-07-02

**Authors:** Charoonsri Chusak, Christiani Jeyakumar Henry, Praew Chantarasinlapin, Varanya Techasukthavorn, Sirichai Adisakwattana

**Affiliations:** 1 Department of Nutrition and Dietetics, Faculty of Allied Health Sciences, Chulalongkorn University, Bangkok 10330, Thailand; charoonsri.c@gmail.com (C.C.); praewchan@yahoo.com (P.C.); varanya.te@gmail.com (V.T.); 2 Clinical Nutrition Research Centre, Singapore Institute for Clinical Sciences (SICS), Agency for Science, Technology and Research, Singapore 117599, Singapore; jeya_henry@sics.a-star.edu.sg; 3 Department of Biochemistry, Yong Loo Lin School of Medicine, National University of Singapore, Singapore 117596, Singapore

**Keywords:** *Clitoria ternatea* L. flower extract, in vitro starch digestibility, hydrolysis index, predicted glycemic index

## Abstract

This study aimed to assess the effect of the *Clitoria ternatea* L. flower extract (CTE), on the inhibition of pancreatic α-amylase, in vitro starch hydrolysis, and predicted the glycemic index of different type of flours including potato, cassava, rice, corn, wheat, and glutinous rice flour. The application in a bakery product prepared from flour and CTE was also determined. The results demonstrated that the 1% and 2% (w/v) CTE inhibited the pancreatic α-amylase activity by using all flours as a substrate. Moreover, 0.5%, 1%, and 2% (w/v) CTE showed a significant reduction in the glucose release, hydrolysis index (HI), and predicted glycemic index (pGI) of flour. In glutinous rice flour, 1% and 2% (w/v) CTE had a significantly lower level of rapidly digestible starch (RDS) and slowly digestible starch (SDS) with a concomitant higher level of undigested starch. The statistical analysis demonstrated strong positive significant correlations between the percentage of CTE and the undigested starch of wheat and cassava. The addition of 5%, 10%, and 20% (w/w) CTE significantly reduced the rate of starch digestion of the wheat bread. The pGI of bread incorporated with 5% CTE (w/w) was significantly lower than that of the control bread. Our findings suggest that CTE could reduce the starch digestibility, the HI, and pGI of flour through the inhibition of carbohydrate digestive enzymes. Taken together, CTE may be a potent ingredient for the reduced glycemic index of flours.

## 1. Introduction

Carbohydrates are one of three basic macronutrients that produce energy for our body. Nowadays, edible flour enriched with carbohydrates has been made from several parts of plants, including roots, seeds, and germs [1]. Flour is a common ingredient used for foods and desserts, according to different cooking purposes. The excessive and chronic consumption of flour markedly increases the postprandial blood glucose level and leads to excess visceral fat, which increases both insulin resistance and inflammation, and predisposes one to diabetes, hypertension, and cardiovascular diseases [2]. It has been shown that various types of flour contribute to a different rate and degree of starch hydrolysis, resulting in varying degrees of postprandial blood glucose rise [3]. Normally, the glycemic index (GI) is the measure of the immediate effect on the postprandial glucose level after food consumption, by comparing the percentage of incremental glucose area under the curve (iAUC) of a test food with reference to a standard food. However, the in vivo measurement of GI requires the recruitment of human subjects under ethical committee approval, financial supports, and it is time consuming, and all of these reasons led to widespread acceptance of in vitro starch digestibility studies [4]. 

The predicted glycemic index (pGI) is a common technique used to measure the rate of carbohydrate hydrolysis in foods [4]. It has been found that in vitro methods used to classify foods based on their digestion characteristics are similar to the in vivo situation [5]. There is a positive correlation between the in vitro and in vivo glycemic response [4]. In the focus of nutritional aspects, carbohydrate foods with a low pGI value (<55) can be considered as beneficial foodstuff for human health, in terms of the prevention and treatment of the metabolic syndromes, diabetes, and cardiovascular diseases [6,7]. Replacing or mixing flours with other ingredients such as fruits and vegetables is one of the alternative approaches to reduce the pGI in carbohydrate foods [8]. For example, pomelo containing polyphenols incorporated into bread could lower the predicted glycemic index probably by inhibiting the activity of the carbohydrate hydrolyzing enzymes [9]. It is becoming clear that plant-based ingredients containing polyphenols delay the action of carbohydrate digestive enzymes and thereby reduce the absorption rate of glucose [10,11]. Previously, polyphenols from the extracts of pomegranate, cranberry, grape, and cocoa could bind to the digestive enzymes (α-amylase and glucoamylase), resulting in the inhibition of starch hydrolysis [12]. Therefore, the addition of plant-based ingredients may be capable of reducing the glycemic index during starch hydrolysis.

*Clitoria ternatea* L., commonly known as Butterfly pea, is a plant species belonging to the Fabaceae family. This plant is widely distributed in tropical zones such as Asia, the Caribbean, and Central and South America. In traditional Ayurvedic medicine, *Clitoria ternatea* L. has been used for treating stress and depression and enhancing memory [13]. There have been many pharmacological activities reported for this plant, such as antidiabetic [14], antipyretic [15], anti-inflammatory [16], and antimicrobial activity [17]. *Clitoria ternatea* has been reported to contain rutin, kaempferol, delphinidin, and related glycosides [18]. Our recent reports demonstrated that an aqueous extract of CTE inhibited the activity of carbohydrate digestive enzymes such as intestinal α-glucosidase and pancreatic α-amylase [19]. In addition to the biological pigment, the flower of *Clitoria ternatea* has been used as a colorant in various foods, beverages, and desserts in Asia. This colorant flower is regularly mixed with rice, bread, cookies, flours, and other traditional foods and desserts with a variety of ratios. For example, some traditional Thai desserts are made using cassava flour or glutinous rice flour mixed with butterfly pea juice at various concentrations to color and are then steamed until cooked. Moreover, sticky rice noodles are made by the mixture of rice flour with CTE juice. Although the pancreatic α-amylase and α-glucosidase inhibitory activity of CTE is well-documented, studies regarding its effect of pancreatic α-amylase action and in vitro starch digestibility using various types of flour have not been taken. Particularly, the potential food application of CTE in flour-based products remains unknown. Therefore, the aim of the present study was to investigate the effect of *Clitoria ternatea* L. flower extract on the activity of pancreatic α-amylase, in vitro starch hydrolysis, and predicted glycemic index of potato, cassava, rice, corn, wheat, and glutinous rice flour. The application in bread prepared from wheat flour and CTE was also determined.

## 2. Materials and Methods 

### 2.1. Chemicals and Reagents

Commercial flours including potato, rice, glutinous rice, wheat, corn, and cassava flours were purchased from a supermarket. Porcine pancreatic α-amylase Type VI-B (catalogue number: A3176) and 3,5-dinitrosalicylic acid were purchased from the Sigma-Aldrich Chemical Co., Ltd. (St. Louis, MO, USA). Amyloglucosidase was obtained from Roche Diagnostics (Indianapolis, IN, USA). The glucose oxidase-peroxidase (GOPOD) kit was purchased from HUMAN GmbH (Wiesbaden, Germany).

### 2.2. Extraction

The dried flower of *Clitoria ternatea* L. was purchased from a local herbal drug store, Bangkok, Thailand. The extraction was performed according to a previous study [20]. The content of the phenolic compounds and the total anthocyanins in the CTE was 53.08 ± 0.08 mg gallic acid equivalents/g extract and 1.08 ± 0.12 mg delphinidin-3-glucoside equivalents/g extract, respectively.

### 2.3. Preparation of Flour and Extract

Flour (0.25 g) was dissolved with 50 mL of boiled water at 100 °C and stirred for 10 min. The flour solution was allowed to cool at room temperature for 10 min. Furthermore, the CTE powder was dissolved in 0.1 M phosphate buffer saline (PBS) or 0.2 M sodium acetate buffer. The CTE solution was vortexed for 10 min. The CTE solution was added into the flour solution (final concentration: 0.5%, 1%, and 2% w/v) and subjected to in vitro digestion.

### 2.4. Inhibition of Pancreatic α-Amylase 

The activity of pancreatic α-amylase was carried out using a modified procedure of Adisakwattana et al. [19]. Fifty microliters of the flour solution were mixed with 100 µL of CTE in 0.1 M phosphate buffer saline (PBS). Fifty microliters of porcine pancreatic α-amylase (15 U/mL) in 0.1 M PBS, pH 6.9, was then added and the mixture was made to 250 µL with 0.1 M PBS. After incubation at 37 °C for 10 min, the reaction was terminated by adding 250 µL of DNS reagent (1% DNS, 0.2% phenol, 0.05% Na_2_SO_3_, and 1% NaOH in distilled water) and heated at 100 °C for 10 min. Then, 40% potassium sodium tartrate (250 µL) was added to stabilize the color. After cooling at room temperature, the absorbance was measured at 540 nm. The pancreatic α-amylase inhibitory activity was calculated as the percentage inhibition. A control was prepared using the same procedure, replacing the CTE solution with 0.1 M PBS.

### 2.5. *In Vitro* Starch Digestibility and Predicted Glycemic Index (pGI)

The in vitro digestion of flour and CTE was performed according to a previous method with some modifications [21,22]. Fifty microliters of the flour solution were mixed with 100 µL of CTE in 0.2 M sodium acetate buffer. The mixture was incubated with 50 µL of porcine pancreatic α-amylase (15 U/mL) and 50 µL of amyloglucosidase (31.25 µg/mL) in 0.2 M sodium acetate buffer, pH 6.0, at 37 °C for 180 min. After heating at 100 °C for 10 min, for stopping the reaction, the supernatant was measured for the glucose content using a glucose oxidase-peroxidase (GOPOD) kit. The values were plotted a graph and the area under the curve (AUC) was calculated using the trapezoidal rule. The hydrolysis index (HI) was calculated from the percentage of the area under the hydrolysis curve of the sample to the area under the curve of the standard glucose. The predicted glycemic indices (pGI) of the samples were estimated according to the followed equation: pGI = 39.71 + 0.549 HI [4]. A control was prepared using the same procedure, replacing the CTE solution with 0.2 M sodium acetate buffer.

### 2.6. Estimation of Starch Fraction

The starch fraction was calculated based on the in vitro starch digestibility of samples. Rapidly digestible starch (RDS) was calculated as the amount of glucose present in the sample at 20 min of the in vitro digestion, whereas slowly digestible starch (SDS) was calculated as the difference between the amount of glucose measured at 120 min and 20 min [21,23]. The undigested starch was calculated as the amount of glucose that was not digested within 120 min. The conversion factor from the glucose to starch was 0.9.

### 2.7. Bread Preparation

Wheat flour and other dry ingredients as a % on the weight of flour basis, including sugar (5%), salt (1.8%), yeast (3%), Benecel methylcellulose, and hydroxypropylmethylcellulose (2%) (Ashland, Covington, KY, USA), were mixed using a Kitchen-Aid bowl mixer at speed 53 rpm for 1 min. The levels of CTE incorporated into this formulation were 5%, 10%, and 20% (w/w) of the wheat flour basis, by adding to mix with other dry ingredients. Based on the wheat flour, vegetable oil (6%), white egg (40%), and milk (70%) were added and then mixed together at speed of 160 rpm for 10 min. After that, the batter was poured into a mold, placed at 30 °C and 90% relative humidity for 50 min, and baked at 150 °C for 40 min. The loaf was removed from the mold and cooled at room temperature. The bread sample was packed in a sealed polyethylene bag until analysis. After baking, the in vitro starch hydrolysis of the bread with or without CTE was determined to indicate the in vitro starch digestibility and pGI. The bread samples were weighed (1 g) and mixed with distilled water (10 mL) while stirring for 10 min. The in vitro starch digestion of the bread was performed according to the above-mentioned method. A control bread was prepared using the same procedure, without the CTE.

### 2.8. Statistical Analysis

The data were expressed as mean ± standard error of the mean (SEM). The statistical significance of the results was evaluated by one-way ANOVA using Duncan multiple comparisons, using SPSS version 22.0 (SPSS Inc., Chicago, IL, USA). The Pearson’s correlation coefficients (r) were calculated between the CTE concentration and undigested starch. *p* < 0.05 was considered statistically significant.

## 3. Results

### 3.1. Inhibition of Pancreatic α-Amylase 

**Figure 1:**
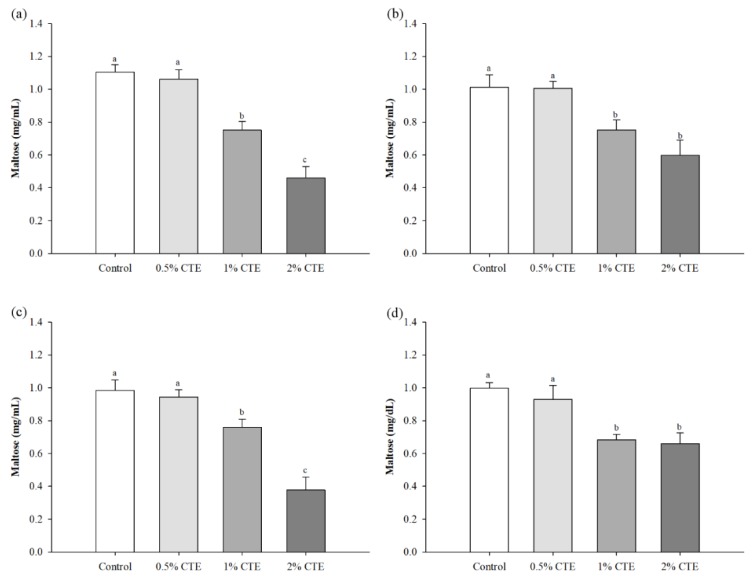

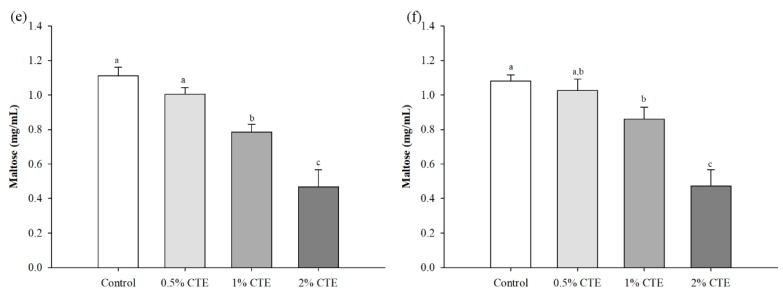
The amount of maltose released from (**a**) potato flour, (**b**) rice flour, (**c**) glutinous rice flour, (**d**) wheat flour, (**e**) corn flour, and (**f**) cassava flour when combination with the different concentrations of CTE against pancreatic α-amylase activity at 10 min. The results are expressed as mean ± standard error of the mean (SEM), *n* = 4. The different letters denote statistically significant differences in mean values. (*p* < 0.05) Mean values with the same superscript letters (a or b) were similar and no statistically significant differences were observed for these samples. CTE—*Clitoria ternatea* L. flower extract.

As shown in Figure 1, the amount of maltose released from all of the flours was observed after 10 min of incubation. There was a significant reduction for the release of maltose after mixing the potato, rice, glutinous rice, wheat, corn, and cassava flour with 1% and 2% (w/v) CTE, compared with the control (*p* < 0.05). 

The percentage of pancreatic α-amylase inhibitory activity after mixing the CTE into flour is shown in Table 1. The increased percentage of pancreatic α-amylase inhibitory activity was concomitant with the increased concentration of CTE. The results demonstrated that the mixture of potato, rice, glutinous rice, wheat, corn, and cassava flours with 1% and 2% (w/v) CTE resulted in a higher pancreatic α-amylase inhibitory activity than that of the 0.5% (w/v) of CTE (*p* < 0.05). At 2% (w/v) CTE, the potato flour had the highest percentage of pancreatic α-amylase inhibitory activity, followed by glutinous rice, rice, wheat, corn, and cassava, respectively.

### 3.2. In Vitro Starch Digestibility and Predicted Glycemic Index (pGI)

**Figure 2:**
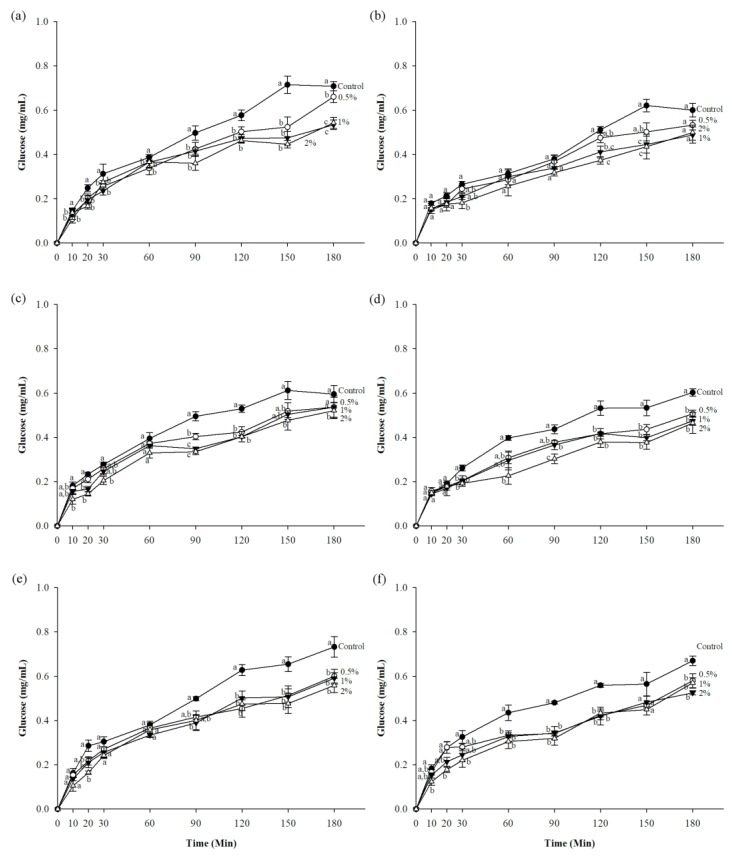
The amount of glucose released from (**a**) potato flour, (**b**) rice flour, (**c**) glutinous rice flour, (**d**) wheat flour, (**e**) corn flour, and (**f**) cassava flour when in combination with the different concentrations of CTE under in vitro digestibility during 180 min. The value of 0.5%, 1%, and 2% (w/v) represent the concentration of CTE, respectively. The results are expressed as mean ± standard error of the mean (SEM), *n* = 4. The different letters denote statistically significant differences in mean values. (*p* < 0.05) Mean values with the same superscript letters (a, b or c) were similar and no statistically significant differences were observed for these samples.

The results of in vitro starch digestibility of flour at different concentration of CTE are shown in Figure 2. The incorporation of CTE decreased the glucose released from flour. The results demonstrated that the amount of glucose released from the mixture of CTE and flours was lower than the control. The addition of 2% (w/v) CTE into flour significantly caused the highest inhibition of starch digestibility. 

Table 2 represents an interpretation of the hydrolysis index (HI) and the predicted glycemic index (pGI) of all of the samples. The HI and pGI of potato, rice, glutinous rice, wheat, corn, and cassava flour with 0.5%, 1%, and 2% (w/v) of CTE were significantly lowered when compared with the control (*p* < 0.05). Interestingly, the addition of 2% (w/v) CTE caused the reduction of pGI of the glutinous rice, wheat, and cassava flour from the high value to the medium value (GI < 70).

### 3.3. Starch Fraction

**Figure 3:**
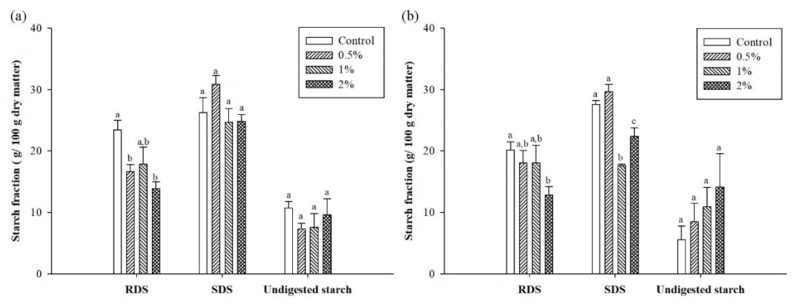

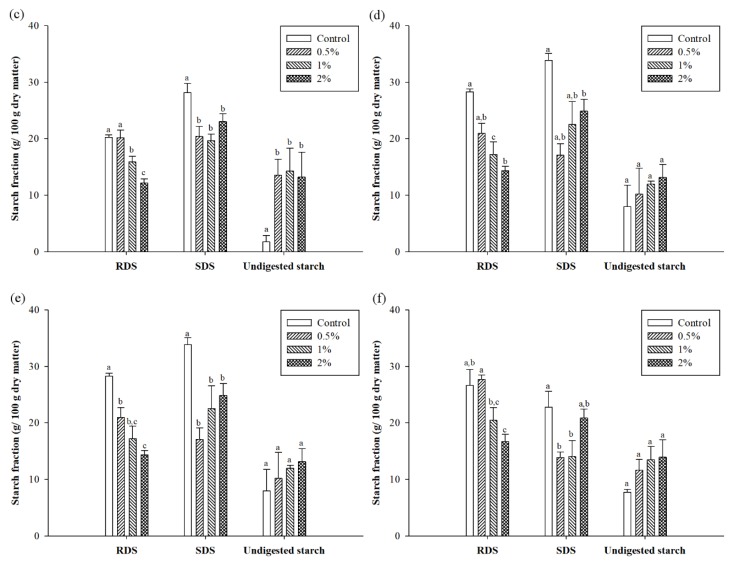
Starch fraction after in vitro digestibility of (**a**) potato flour, (**b**) rice flour, (**c**) glutinous rice flour, (**d**) wheat flour, (**e**) corn flour, and (**f**) cassava flour when in combination with the different concentration of CTE. The value of 0.5%, 1%, and 2% (w/v) represent the concentrations of CTE, respectively. The results are expressed as mean ± standard error of the mean (SEM), *n* = 4. The different letters denote statistically significant differences in mean values. (*p* < 0.05) Mean values with the same superscript letters (a, b or c) were similar and no statistically significant differences were observed for these samples. RDS: rapidly digestible starch; SDS: slowly digestible starch.

The RDS, SDS, and undigested starch of flours are presented in Figure 3. The RDS content of six flours mixed with at 2% (w/v) CTE significantly decreased when compared with the control (*p* < 0.05). The addition of CTE at 0.5%, 1%, and 2% (w/v) caused a reduction in the SDS content of glutinous rice, corn, and cassava flour (*p* < 0.05). However, the CTE did not alter the SDS content of potato flour. The observed results also found that only the glutinous rice flour significantly increased the undigested starch with the addition of CTE (*p* < 0.05). Table 3 shows the correlation between the concentration of CTE and undigested starch of flour. The undigested starch of the wheat and cassava flour correlated significantly and positively with the concentration of CTE (*r* = 0.650 and 0.758, respectively; *p* < 0.05). However, no significant correlation was observed between the concentration of CTE and other flours, including potato flour, rice flour, glutinous rice flour, and corn flour.

### 3.4. In Vitro Digistibility of Bread

**Figure 4:**
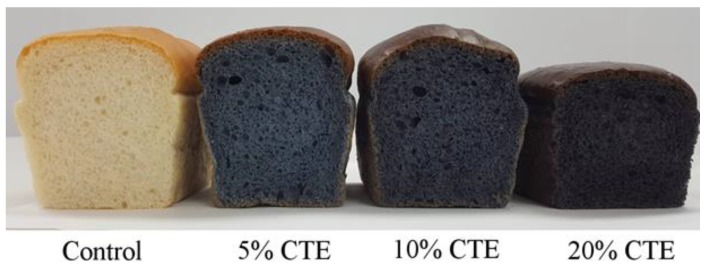
The cross section of bread made from wheat flour and CTE.

**Figure 5:**
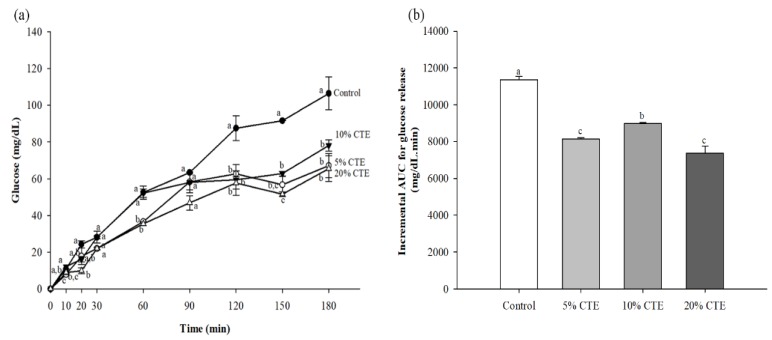
The amount of glucose released from wheat bread after in vitro digestibility (**a**) and incremental area under the curve (iAUC) for glucose release (**b**) when in combination with the different concentrations of CTE. The values of 5%, 10%, and 20% (w/w) represent the concentration of CTE, respectively. The results are expressed as mean ± standard error of the mean (SEM), *n* = 4. The different letters denote statistically significant differences in mean values. (*p* < 0.05) Mean values with the same superscript letters (a, b or c) were similar and no statistically significant differences were observed for these samples.

Cross sections of bread made from wheat flour and CTE are shown in Figure 4. The in vitro starch digestibility of wheat bread with 5%, 10%, and 20% (w/w) of the CTE are presented in Figure 5a. The amount of glucose released from the bread with CTE was lower than that of the control. The addition of 5%, 10%, and 20% (w/w) CTE into wheat bread significantly reduced the rate of starch digestion after 120, 150, and 180 min of incubation (*p* <0.05). As shown in Figure 5b, the iAUCs for the glucose release of bread incorporated with 5–20% (w/w) CTE were 8136 ± 82, 8997 ± 42, and 7363 ± 386 mg/dL·min, respectively (the control = 11,364 ± 172 mg/dL·min). The pGI of the bread with 5–20% (w/w) CTE was 65.40 ± 0.26, 68.11 ± 0.13, and 62.96 ± 1.22, respectively, whereas the wheat bread had the pGI of 75.58 ± 0.54. 

## 4. Discussion

Starchy foods or ingredients are digested by amylolytic α-amylases and α-glucosidase enzymes, including maltase-glucoamylase and sucrase-isomaltase, at the brush border of the small intestine [24]. Thereafter, the absorbable glucose is transported into the bloodstream through the glucose transporter in the small intestine. The high rate of digestion and absorption of these foods contributes to a rise in postprandial glucose, related to health consequences. The physio-chemical properties of carbohydrate foods are normally investigated by measurement of the rate and extent of glucose release after enzymatic digestion under controlled conditions [25]. Our findings demonstrated that a higher amount of maltose and glucose released from flours was observed after in vitro digestion. When the CTE was mixed with the flours, the release of maltose and glucose was significantly decreased. These findings suggest that CTE has a potential to reduce the release of maltose and glucose from flours, leading to a delay in the rate of starch digestibility. In agreement with another study, CTE inhibited the pancreatic α-amylase and intestinal α-glucosidase related to its phytochemical compounds [26]. It has been revealed that the phytochemical compounds in CTE are delphinidin-3, 5-glucoside, delphinidin-3-glucoside, malvidin-3-glucoside, delphinidin-based ternatins (ternatins A1–A3, B1–B4, C1–C5, and D1–D3), kaempferol, quercetin-3-*O*-(2-rhamnosyl) rutinoside, and rutin [18]. In particular, rutin and kaempferol could inhibit the pancreatic α-amylase and intestinal α-glucosidase activity [27,28]. Moreover, the natural delphinidin and malvidin compounds have shown a competitive inhibiting effect against the intestinal α-glucosidase [29,30]. A study conducted by Podsędek et al. [31] observed that the degree of the inhibitory effect on the carbohydrate digestive enzymes is positively correlated with the concentration of anthocyanins. We suggest that the phytochemical compounds in CTE may contribute to delaying the hydrolysis of starch by inhibiting the carbohydrate digestive enzymes, including pancreatic α-amylase and intestinal α-glucosidase. Additionally, Zhu et al. [32] also explained other mechanisms of polyphenols on delayed starch digestibility. It found that polyphenol could interact with the starch chains to form the complex, resulting in the alteration of enzyme susceptibility. This evidence was supported from the higher content of undigested starch after in vitro digestion of starch and anthocyanins in blue maize [33]. The interaction between polyphenols and starch was due to the non-covalent bonding and/or the hydrogen binding formation [34]. Further studies are needed to define the hypothesized mechanisms specific to the interaction between the anthocyanins in the CTE and flours, and the type of enzyme inhibition. 

For nutritional purposes, the starch in food is generally classified into three categories, including rapidly digestible starch (RDS), slowly digestible starch (SDS), and resistant starch (RS) [35]. In terms of RDS, starch is readily and completely digested in the small intestine associated with more rise postprandial plasma glucose within first 20 min. SDS is a complete digestion of starch in the small intestine with slow rate [5]. Furthermore, RS is defined as the dietary starch that resists digestion in the small intestine. According to a previous study [36], undigested starch contributes to the resistant starch, which is due to the inhibitory activity of α-amylase by antinutrients (e.g., polyphenols). The various amount of starch fractions in the different types of flour depends on their physical and chemical characteristics [37]. The observed results indicate that all flours had a higher level of RDS with a concomitant lower content of undigested starch. The gelatinization is one of the factors affecting starch hydrolysis during the cooking process [38]. Thus, the gelatinization of the starch granule induces an increase in the RDS response with the release of the glucose molecule. The results also showed that the flour with a high RDS content produced a significantly higher level of HI and pGI, whereas the higher SDS content with lower RDS reduced the levels of HI and pGI. A previous study supported our findings, indicating that the pGI value was correlated with the parameters of the digestible starch fractions, including RDS and SDS. In particular, SDS is found to be the main contributing factor to the GI [39]. It has been reported that an intake of diet containing a high RDS level could induce a rapid hyperglycemic response and a subsequent glucose-induced insulin secretion from pancreatic β-cells [5]. In contrast, undigested starch (RS) in human diets provides functional properties and applications for delaying postprandial glucose [40] and improving postprandial insulin [41]. Our findings demonstrated that the addition of CTE into glutinous rice, wheat, corn, and cassava flour causes the reduction of RDS and SDS, in relation to the increased content of undigested starch. Moreover, the undigested starch of wheat and cassava flours significantly and strongly correlated with the concentration of CTE. For traditional use, glutinous rice flour, ground from glutinous rice or sticky rice, is usually used as the ingredient for desserts, sweets, rice cakes, and puffed rice in Asia and Southeast Asia [42]. Basically, glutinous rice has been classified as high GI because of its high amylopectin content and rate of digestion [43]. The starch digestibility of glutinous rice produces more rapidly and is more complete than other high-amylose rice varieties. Chan et al. [44] found an increased glycemic response and GI values in Caucasian and Asian populations after the consumption of glutinous rice, which was similar to a previous study of Ranawana et al. [45]. In the current study, the mixture of CTE into glutinous rice flour can reduce the pGI value, suggesting that CTE suppresses the digestive process of glutinous rice flour to absorbable monosaccharides. A combination of glutinous rice flour and CTE might have opportunities for flour applications to reduce the GI of the food products. 

Several studies have reported that the plant-based diets containing polyphenols alter the glycemic index of various foods. The current study found that the addition of CTE caused the rate of carbohydrate digestion and pGI of wheat bread to slow down. Our results are in agreement with Reshmi et al. [9], who reported the in vitro glycemic impact of bread fortified with pomelo fruit. Because of the action of phenolic compounds and the flavonoids in pomelo, the bread fortification with pomelo caused a lower level of digestible starch with a concomitant increased level of undigested starch. Lemlioglu-Austin et al. [46] also found that the incorporation of phenolic-rich sorghum bran extract into porridges contributes to slow starch digestion with a reduced GI and increased undigested starch. In addition, thermal processing also affects starch digestibility by the alteration of its granular structure [47]. The cooking process with heating and excess water induces the gelatinization of the starch granule, increasing in starch digestibility. The crumb portions of baked bread increased the starch digestibility when compared with the crust portion, because the starches in the crust portion are not completely gelatinization after baking. The fortification of green tea polyphenol in baked bread reduced in glucose release for both the crumb and crust after in vitro digestion [48] and reduced the rapidly digestible starch of white bread samples [49]. During baking, the interaction between gelatinized starch granules and the gluten network occurs in crumb, causing a loss of kinetic energy and a subsequent increase in firmness [50]. Previous evidence revealed that polyphenols can form a complex with bread ingredients including protein and polysaccharides [50]. The formation of polyphenols and polysaccharides or protein as enzymes clearly indicates a reduced in vitro digestibility [34]. Moreover, polyphenols affect the breadmaking quality by altering the flour protein properties [51]. The interaction of polyphenols and gluten proteins in wheat bread is associated with a reduction in protein cross-linking, resulting in decreased bread volume. Our study showed that the addition of CTE caused the bread volume to and air pocket in wheat bread to reduce. These findings are consistent with a study of Pathak et al., who reported that the addition of mango fruit peel powder could decrease the volume and height of loaf, whereas it increased the density of the loaf with less visible air pockets of bread, owning to the compact crumb structure [52]. It could be explained that the gluten network was not completely formed, leading to the ineffectiveness to hold air during fermentation, which caused the decreased loaf volume [53]. According to our findings, the addition of CTE into various types of flours successfully altered the parameters of starch digestibility and consequently decreased the level of HI and pGI. Further studies are warranted to elucidate whether the consumption of bread incorporated with CTE delays postprandial glucose in humans. 

## 5. Conclusions

The present study demonstrated that CTE could inhibit pancreatic α-amylase activity, leading to a reduction of maltose release from flour. The addition of CTE to flour alters in vitro starch digestibility, resulting in a reduction in the amount of glucose released from the various types of flour and a subsequent reduction of HI and pGI. Moreover, the addition of the CTE reduced the starch digestibility rate and pGI of wheat bread. The findings indicate that the addition of CTE into flour can inhibit the starch digestibility of flour through the inhibition of carbohydrate digestive enzymes, including pancreatic α-amylase and intestinal α-glucosidase. We suggest that CTE may be a potent ingredient for the reduced glycemic index of flours.

## Figures and Tables

**Table 1:** The percentage of pancreatic α-amylase inhibitory activity of CTE.

CTE	% Inhibition					
	Potato	Rice	Glutinous Rice	Wheat	Corn	Cassava
0.5% (w/v)	7.1 ± 4.7	23.8 ± 9.2	20.8 ± 9.2	17.4 ± 6.5	24.1 ± 9.5	12.9 ± 1.4
1% (w/v)	56.7 ± 7.8	82.1 ± 8.1	51.7 ± 8.5	50.1 ± 7.7	48.3 ± 8.2	34.5 ± 8.9
2% (w/v)	93.4 ± 5.7	87.3 ± 13.4	89.7 ± 11.9	85.2 ± 4.3	81.6 ± 10.6	79.9 ± 19.7

The results are expressed as mean ± standard error of the mean (SEM), *n* = 4. CTE—*Clitoria ternatea* L. flower extract.

**Table 2:** The hydrolysis index (HI) and predicted glycemic index (pGI) of the flours with CTE.

**CTE**	**Hydrolysis Index (HI)**					
	**Potato**	**Rice**	**Glutinous rice**	**Wheat**	**Corn**	**Cassava**
Control	85.2 ± 0.5 ^a^	74.1 ± 4.3 ^a^	86.9 ± 2.4 ^a^	74.3 ± 2.5 ^a^	94.4 ± 3.6 ^a^	81.0 ± 1.3 ^a^
0.5% (w/v)	71.0 ± 1.6 ^b^	63.4 ± 2.4 ^a,b^	65.5 ± 4.1 ^b^	62.4 ± 3.7 ^b^	69.5 ± 3.2 ^b^	65.5 ± 2.2 ^b^
1% (w/v)	68.8 ± 3.7 ^b,c^	57.2 ± 4.9 ^b^	63.7 ± 5.2 ^b^	61.0 ± 1.4 ^b^	68.5 ± 2.2 ^b^	61.3 ± 3.4 ^b,c^
2% (w/v)	62.2 ± 2.1 ^c^	55.2 ± 3.3 ^b^	50.3 ± 5.1 ^c^	50.4 ± 4.8 ^c^	59.0 ± 2.3 ^c^	51.5 ± 6.0 ^c^
**CTE**	**Predicted Glycemic Index (pGI)**					
	**Potato**	**Rice**	**Glutinous rice**	**Wheat**	**Corn**	**Cassava**
Control	86.5 ± 0.3 ^a^	80.4 ± 2.4 ^a^	87.2 ± 1.3 ^a^	80.5 ± 1.4 ^a^	91.2 ± 2.0 ^a^	84.2 ± 0.7 ^a^
0.5% (w/v)	78.7 ± 0.9 ^b^	74.5 ± 1.3 ^a,b^	75.7 ± 2.2 ^b^	74.0 ± 2.0 ^b^	77.9 ± 1.8 ^b^	75.7 ± 1.2 ^b^
1% (w/v)	77.5 ± 2.0 ^b,c^	71.1 ± 2.7 ^b^	74.7 ± 2.8 ^b^	73.2 ± 0.8 ^b^	77.3 ± 1.2 ^b^	73.4 ± 1.9 ^b,c^
2% (w/v)	73.8 ± 1.1 ^c^	70.0 ± 1.8 ^b^	67.4 ± 2.8 ^c^	67.4 ± 2.6 ^c^	72.1 ± 1.3 ^c^	68.0 ± 3.3 ^c^

The results are expressed as mean ± standard error of the mean (SEM), *n* = 4. The different letters denote statistically significant differences in mean values. (*p* < 0.05) Mean values with the same superscript letters (a, b, or c) were similar and no statistically significant differences were observed for these samples.

**Table 3:** Correlation coefficients calculated between the concentrations of *Clitoria ternatea* (CTE) and undigested starch contents after in vitro digestion.

	Potato	Rice	Glutinous Rice	Wheat	Corn	Cassava
**CTE**	−0.040	0.511	0.486	0.650 *	0.373	0.758 *

* Significant correlations (*p* < 0.05).
